# TCAB1: a potential target for diagnosis and therapy of head and neck carcinomas

**DOI:** 10.1186/1476-4598-13-180

**Published:** 2014-07-28

**Authors:** Chong-kui Sun, Xiao-bo Luo, Ya-ping Gou, Ling Hu, Kun Wang, Chao Li, Zhen-ting Xiang, Ping Zhang, Xiang-li Kong, Chao-liang Zhang, Qin Yang, Jing Li, Li-ying Xiao, Yan Li, Qian-ming Chen

**Affiliations:** 1 State Key Laboratory of Oral Diseases, West China Hospital of Stomatology, Sichuan University, Chengdu, Sichuan 610041, China; 2 Department of Head and Neck Surgery of Sichuan Cancer Hospital, Chengdu, Sichuan 610041, China; 3 Division of Radiation and Cancer Biology, Department of Radiation Oncology, Washington University in Saint Louis, School of Medicine, 4511 Forest Park, St. Louis, MO 63108, USA

**Keywords:** TCAB1, Head and neck cancers, Proliferation, Invasion, Biomarker

## Abstract

**Background:**

WRAP53, including α, β and γ isoforms, plays an important role not only in the stability of p53 mRNA, but also in the assembly and trafficking of the telomerase holoenzyme. It has been considered an oncogene and is thought to promote the survival of cancer cells. The aim of this study was to detect the role of TCAB1 (except WRAP53α) in the occurrence and development of head and neck carcinomas.

**Methods:**

Immunohistochemistry was used to detect the TCAB1 expression in clinical specimen sections and performed western blotting to check the TCAB1 expression levels in cell lines. TCAB1 was depleted using shRNA lentivirus and the knockdown efficiency was assessed using q-PCR and Western blotting. We performed CCK-8 assays and flow cytometry to check the cell proliferation potential and used the trans-well assay to test the invasion ability *in vitro*. Xenografts were used to detect the tumor formation potential *in vivo*. Moreover, we performed cDNA microarray to investigate the candidate factors involved in this process.

**Results:**

We observed a notable overexpression of TCAB1 in head and neck carcinoma clinical specimens as well as in carcinoma cell lines. Knockdown of TCAB1 decreased the cellular proliferation potential and invasion ability *in vitro*. cDNA microarray analysis suggested the possible involvement of several pathways and factors associated with tumorigenesis and carcinoma development in the TCAB1-mediated regulation of cancers. Furthermore, the xenograft assay confirmed that the depletion of TCAB1 would inhibit tumor formation in nude mice. The immunohistochemistry results of the mice tumor tissue sections revealed that the cells in shTCAB1 xenografts showed decreased proliferation potential and increased apoptotic trend, meanwhile, the angiogenesis was inhibited in the smaller tumors form shTCAB1 cells.

**Conclusions:**

Our study demonstrated that depletion of TCAB1 decreased cellular proliferation and invasion potential both *in vitro* and *in vivo*. The data indicated that TCAB1 might facilitate the occurrence and development of head and neck carcinomas. In future, TCAB1 might be useful as a prognostic biomarker or a potential target for the diagnosis and therapy of head and neck carcinomas.

## Background

Telomerase is a ribonucleoprotein (RNP) containing RNA and proteins, and was first discovered in 1985 in the ciliate Tetrahymena cell by Greider and Blackburn [1]. Most human tumor cells, about 85%-90%, utilize the telomerase holoenzyme to maintain telomere length by adding TTAGGG repeats to telomere ends [2]. In the last 10 years of 20^th^ century, several labs had investigated the telomerase activity in head and neck tumors. In normal oral mucosa, the frequency of telomerase positive cells was as low as 0%-5%, while approximately 50% and 85%-100% of cells were telomerase positive in premalignant tissues and in malignant head and neck squamous cell carcinoma, respectively [2-9]. Even though previous reports indicated that telomerase played an indispensable role in tumorigenesis and development of head and neck tumors, the detailed underlying mechanism is still poorly understood.

Telomerase Cajal body protein 1(TCAB1), accumulated in Cajal bodies, is seen as a factor associated with dyskerin, which plays a core role in the assembly and stability of telomerase. Further, TCAB1 was verified to be involved in telomerase holoenzyme assembly and in driving telomerase to Cajal body [10,11]. Although a subsequent study indicated that telomerase could recruit TCAB1 and Cajal body independently [12], TCAB1 was regarded as an essential component of the telomerase holoenzyme. Since the localization of telomerase to Cajal body was regarded a crucial step in the telomerase holoenzyme assembly in telomerase positive tumors, mutation of *TCAB1* gene could result in disorders as dyskeratosis congenita associated with telomeres shortening, which partly caused aging senescence or cancer [13,14]. Thus, it is evident that *TCAB1* might be an oncogene which to a certain extend could facilitate tumorigenesis and tumor development.

Although the involvement of TCAB1 in telomere maintenance was uncovered only recently in 2009 [10], TCAB1 itself is not a newly discovered protein, and was well known prior to this as WRAP53 or WDR79. WRAP53 could transcribe several different isoforms, as WRAP53α, β, or γ, and the previous studies demonstrated that only the WRAP53α was identified as a natural antisense transcript of p53, which regulates p53 protein levels by targeting the 5’- untranslated region of p53 mRNA [15]. So here we used TCAB1 to represent the WRAP53 isoforms except α. Previously reported data *in vitro* suggested that overexpression of WRAP53 could induce cellular transformation while WRAP53 knockdown by exogenous siRNA on the other hand, could induce cellular apoptosis [16], also indicating potential oncogenic characteristics of TCAB1. Head and neck carcinoma is approximately the sixth most common cancer among global cancers [17,18], and the five year survival rate has remained at about 50% in the past decades [19]. These cancers are indeed harmful to humans, but to date, there are no specific studies on the functional relevance of TCAB1 in head and neck tumors.

Our findings showed that TCAB1 (except WRAP53α) was overexpressed in human head and neck carcinoma cell lines, including human nasopharyngeal carcinoma (NPC) cell line CNE1, oral squamous carcinoma cell (OSCC) lines HSC-3, Cal-27, and adenoid cystic carcinoma (ACC) cell line ACC2. Meanwhile, TCAB1 was overexpressed in most (~80%) specimens from nasopharyngeal carcinoma patients compared to the nasopharyngitis patients, while was expressed at low levels in human primary normal oral cells, human periodontal ligament cells (PDLC) and dental pulp cells (DPC) (*P* < 0.05). Further studies showed that knockdown of TCAB1 by exogenous shRNA using lentivirus reduced the proliferation potential of human OSCC cell lines Cal-27, HSC-3 and ACC cell line ACC2 *in vitro*, and the cells treated with shTCAB1 lentivirus generated smaller tumors in nude mice. Moreover, we performed immunohistochemistry (IHC) of the xenografts tumor sections from shTCAB1 treated OSCC cells compared to the shScrambled cells. The data showed that the proliferation markers, both the Ki-67 and PCNA, were lower expressed while the apoptotic markers were upper regulated, which suggested depletion of TCAB1 could also inhibit the growth of cancer cells *in vivo*. The cDNA microarray data indicated that the p53 signaling pathway, cell cycle and apoptosis pathway, Jak-STAT signaling pathway and several others, might be involved in this TCAB1-mediated tumor regulation process. All of our studies imply that TCAB1 is potential target for diagnosis of and molecular therapy for head and neck cancers.

## Materials and methods

### Clinical specimens

A total of seventy paraformaldehyde-fixed paraffin-embedded tissues with histopathology reports were collected in order to detect TCAB1 expression. Among these, fifty were NPC tissues and twenty were nasopharyngitis (NPi) tissues. All seventy tissue specimens were procured from the Sichuan Province Tumor Hospital from 2010 to 2012 (NPC: 2011–2012, NPi: 2010–2012). The study was carried out under the approval and supervision of the Ethics Committee of Sichuan University and written informed consents were obtained from each patient.

### Cell lines and cell culture

Two OSCC cell lines, HSC-3, Cal-27, one NPC cell line CNE1, and one ACC cell line ACC2 were used in this study. HSC-3, Cal-27, and ACC2 were cultured in Dulbecco’s modified Eagle’s medium (DMEM, HyClone) supplemented with 10% fetal bovine serum (FBS, HyClone) and penicillin-streptomycin (Thermo Scientific). CNE1 was cultured in RPMI-1640 (HyClone) with 10% fetal bovine serum and penicilin/streptomycin. All of the cell lines were obtained from state key laboratory of oral diseases, and HSC-3 (JCRB0623) was purchased from the cell bank of Japanese Collection of Research Bioresource (JCRB, Shinjuku, Japan). Primary periodontal ligament cells (PDLC) and human dental pulp cells (hDPCs) were isolated from clinical fresh specimens and cultured in DMEM with 10% fetal bovine serum and penicillin-streptomycin. All cells were cultured in a humidified incubator at 37°C with 5% CO2.

### Immunohistochemistry

Pathological specimens were analyzed by routine immunohistochemical methods [20,21]. In brief, 4 μm formalin-fixed and paraffin-embedded (FFPE) sections were de-paraffinized with xylene twice and rehydrated in graded 100%, 90%, 80%, 70% alcohol solution, and antigens were retrieved with Tris-EDTA buffer for 3-5 min at 100°C. The slides were peroxidase blocked with 3% hydrogen peroxide solution for 10 min and then blocked using 5% bovine serum albumin (Sigma) for 30 minutes. Slides were subsequently incubated with primary antibody against TCAB1 (Novus, 1:200), VEGF (Santa cruz biotechnology, 1:100), Bcl-2 (Santa cruz biotechnology, 1:150), Caspase 3 (Santa cruz biotechnology, 1:150), Ki-67 (Abcam, 1:150), PCNA (Cell Signaling Technology, 1:100), CD34 (Abcam, 1:25) for 2 h at 37°C or overnight at 4°C and detected with ChemMate™ EnVision kit (DAKO). The slides were counterstained with haematoxylin, dehydrated with graded alcohol and mounted. Each time for IHC, a section slide was used as negative control, of which the only difference was incubated without primary antibody. Finally, scanned the slides and scored the data with imageScope software (Aperio).

### Lentivirus assembly and infection

The shTCAB1-A ~ D lentivirus was constructed and assembled by ShangHai SBO Medical Biotechnology Co. and infected cells with polybrene (5 μg/mL, Millipore). Four different shTCAB1 lentivirus products were used, and labeled shTCAB1-A ~ D (TCAB1, NM_018081.2, A ~ D: site 645–663,708-726,1452-1470 and 1738–1756, respectively). Another shTCAB1-667 lentivirus was assembled using pCMV-dR8.2 dvpr (Addgene), pCMV-V-SVG (Addgene) and shTCAB1-667 (shRNA, targeting CACCCAACCTGAGAACTTCTT, Sigma Aldrich). The cells infected with lentivirus were selected using puromycin (Thermo). Meanwhile, the shScramble lentivirus (shScra) was used as negative control.

### Western blotting

Cells were trypsinized using 0.025% trypsine-EDTA (Gibco) and collected by centrifugation. Total proteins were isolated using the Radio Immunoprecipitation Assay buffer (RIPA lysis buffer, Beyotime), and western blotting was performed using the routine approach. Primary TCAB1 antibody (Novus) was diluted at 1:2000 in 5% BSA and GAPDH (1:5000, Trevigen) was used as an internal control.

### Quantitative real-time PCR

The cells were collected using 0.025% trypsine-EDTA and the total RNA was extracted with TRIzol regent (Life Technologies). Reverse transcription PCR was performed using Primescript RT reagent Kit (TaKaRa), and further quantitative real-time PCR analysis was performed using SYBR select master mix (Applied Biosystems; TCAB1 primers: Forward, 5’-CATATCTGGGACGCATTCACT-3’, Reverse, 5’-GTTGAAGCCACAGAAGAGCTG-3’). The PCR results were quantified using semi-quantitative method as previously reported [22].

### Cell proliferation and cell cycle assay

The cells were seeded in 96-well plates at a density of 10^3^ per well and Cell Counting Kit-8 assay (CCK-8, Dojindo) was performed to check the proliferation potential. Duplicates sets of 4 wells each were assessed for each time point. After every 24 h post seeding, the absorbance was measured at 450 nm using Thermo Scientific Varioskan Flash (Thermo Scientific).

Cells were collected and washed with PBS, fixed with 70% cold ethanol at 4°C for 2 h, and passed through 70 μm Falcon (BD Biosciences) to obtain a mono-dispersed cell suspension. The mono-dispersed cells were incubated with RNase A at 37°C for 30 min and stained with pripidium iodide (PI) at 4°C for 30 min (Cell Cycle Detection Kit, KeyGEN). Cell cycle assay was performed in a flow cytometer (Beckman).

### Cell invasion assay

The matrigel (BD Biosciences) was diluted with serum-free DMEM at 1:5, and added carefully to the bottom of the trans-well insert (8 μm, BD Biosciences) placed in a 24-well plate without any bubbles. For every group, triplicate were maintained. The trans-well inserts were incubated at 37°C at least 1 h before seeding cells. Cells were detached using 0.025% trypsin-EDTA and washed with PBS, centrifuged and resuspended in serum free DMEM. For each group, 5 × 10^4^ cells were seeded in every trans-well insert, and DMEM supplied with 10% FBS was added in every well of the 24-well plate. After culturing for 24 or 48 h, the cells that adhered in the inserts were carefully wiped and fixed with 70% methanol, and stained with haematoxylin.

### cDNA microarray

Cells from shScramble and shTCAB1 groups were prepared simultaneously, and the cDNA microarray assay was done by Invitrogen Co. (Invitrogen, Shanghai, OS130121002, GSE54224). The assays were performed on the platform of Affymetrix GeneChip Human Gene 1.0 ST Array. The raw data was normalized and further analyzed after log transformation of the fold changes (log 2). The expression level of each gene was compared between the shTCAB1 and shScra groups, and a change of 1.3-folds was set as a threshold to identify differences in gene expression. Finally, we analyzed all of the selected genes with Kyoto Encyclopedia of Genes and Genomes (KEGG) to identify the pathways. The detailed ontology results are shown in Table 1.

**Table 1:** KEGG analysis results

**hsa05200: Pathways in cancer (42)**	AKT3, APC, APPL1, BIRC2, BIRC7, BMP2, CDC42, CDK6, CEBPA, CHUK, CUL2, DAPK1, DCC, E2F3, FAS, FGF17, FGF22, FGF6, FGF9, FLT3, FZD10, FZD3, GLI1, HGF, KITLG, KRAS, MAPK8, MECOM, PDGFRB, PIAS2, PIK3CB, PIK3CG, PLD1, PTGS2, RAF1, RXRG, SHH, SMAD4, SOS1, SPI1, WNT1, WNT5B
**hsa04115: p53 signaling pathway (18)**	ATM, ATR, BBC3, CCNB1, CCNB2, CCND2, CDK1, CDK6, FAS, GADD45A, GADD45B, IGFBP3, MDM4, SERPINE1, SESN1, SESN3, TP53AIP1, ZMAT3
**hsa04110: Cell cycle (20)**	ANAPC4, ATM, ATR, CCNB1, CCNB2, CCND2, CDC23, CDK1, CDK6, CDKN2D, E2F3, E2F5, GADD45A, GADD45B, ORC2, ORC4, ORC5, SMAD4, STAG1, TTK
**hsa04210: Apoptosis (15)**	AIFM1, AKT3, ATM, BIRC2, BIRC7, CASP6, CHUK, FAS, IRAK3, IRAK4, NGF, PIK3CB, PIK3CG, PPP3CB, PRKACG
**hsa04151: PI3K-Akt signaling pathway (40)**	AKT3, CCND2, CDK6, CHUK, COL11A1, COL11A2, COL3A1, COL5A3, CREB3L1, CSF3, FGF17, FGF22, FGF6, FGF9, FOXO3, GNGT1, HGF, IFNA13, IFNAR1, ITGB7, JAK2, JAK3, KITLG, KRAS, NGF, NOS3, NR4A1, PCK1,PDGFRB, PIK3CB, PIK3CG, PKN2, PPP2R2A, PPP2R3A, PPP2R3C, PPP2R5A, RAF1, SOS1, TCL1B, THBS4
**hsa04630: Jak-STAT signaling pathway (29)**	AKT3, CCND2, CISH, CRLF2, CSF3, IFNA13, IFNAR1, IFNW1, IL10RB, IL11RA, IL12B, IL12RB1, IL13RA1, IL20, IL6ST, JAK2, JAK3, LIF, LIFR, PIAS2, PIK3CB, PIK3CG, PIM1, SOCS1, SOCS4, SOS1, SPRED1, SPRED3, STAM2

Differently expressed genes (≥1.3-fold change) between the shTCAB1 and shScra groups.

### Animal study and xenografts tumor formation assay

Four-week-old female BALB/c *nu/nu* mice were purchased from the laboratory animal center of Sichuan University and maintained in the animal facility. All experimental procedures involving animals were done in compliance with institutional and governmental requirements, and approved by the laboratory animal center’s Animal Care and Use Committee. Cells were collected and re-suspended in DMEM. A cell density of 5 × 10^6^ cells from each group in 200 μl DMEM were injected subcutaneously into the left and right neck of the BALB/c nude mice respectively. The tumor growth curves were determined by measuring the tumor size using vernier caliper, and tumor volumes were calculated by the formula l*w*h/2 (mm^3^). All mice were mercifully killed at day 27 and the tumors were removed and weighed. Tumors tissues were washed with PBS and fixed with 4% paraformaldehyde for IHC studies.

### TUNEL assay

The 4 μm FFPE xenografts sections were pre-treated as IHC procedures to remove paraffin and rehydrate. In brief, fixed the slides in 4% formaldehyde in PBS for 15 minutes after washing, and permeabilize the slides with 20 μg/ml Proteinase K solution (Promega) for 10 min at room temperature. Wash and fix again. Then equilibrate with equilibration buffer (promega) for 10 minutes at room temperature, and label the slides with TdT reaction mix (promega) for 1 h at 37°C in a humidified chamber. Stop reaction with 2X SSC and wash, counterstain use Vectashield Mounting Medium with DAPI (Vector labs).

### Statistical analysis

Statistical Package for Social Science (SPSS) version 19.0 for windows and GraphPad Prism 6 were used to analyze the data. Student’s *t* test was used to compare the data between every two groups respectively. For all statistical analysis, *P* value less than 0.05 was considered statistically significant.

## Results

### TCAB1 is overexpressed in human head and neck carcinomas cell lines

**Figure 1:**
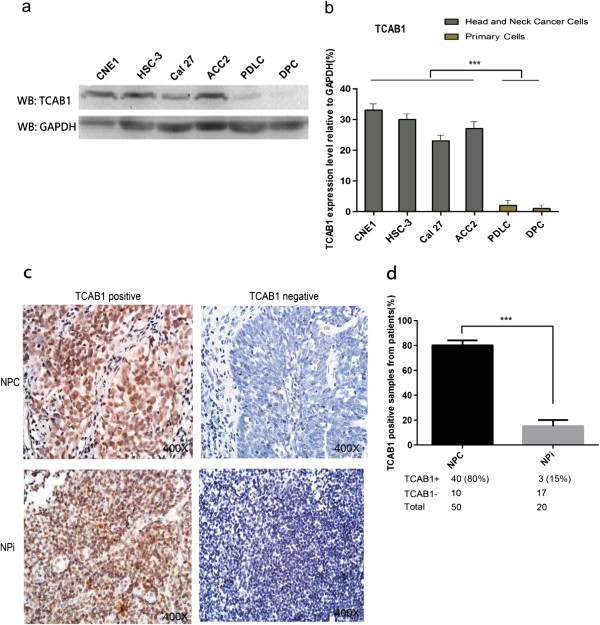
**TCAB1 was overexpressed in cell lines and in tissues of head and neck cancers. a**. Protein level of TCAB1 in head and neck cancer cell lines (the first 4 samples) compared to human normal primary cells (the last 2 samples). **b**. Quantitative results of the TCAB1 protein levels relative to GAPDH in each cell line. **c**. The typical IHC results (Left: TCAB1 positive, Right: TCAB1 negative) of TCAB1 in human NPC and NPi. **d**. Percentage of TCAB1 positive specimens of NPC and NPi in each group. Three different persons estimated the 70 specimens independently, the TCAB1 negative or week expressed specimens were thought as negative here. Statistical analysis was determined by Student’s *t* test (***P < 0.005).

To investigate whether TCAB1 exhibits oncogenic characteristics in human head and neck carcinomas, we checked the protein expression level of TCAB1 in several human head and neck carcinoma cell lines and two normal primary cells. The results indicated that TCAB1 was significantly overexpressed in NPC cell line CNE1, OSCC cell lines HSC-3 and Cal-27, and also in ACC cell line ACC2, while the expression level was relatively low in human primary normal cells, including human periodontal ligament cells (PDLC) and human dental pulp cells (DPC) (Figure 1a, b). Furthermore, we performed immunohistochemistry (IHC) experiments with 50 human NPC specimens and 20 human nasopharyngitis (NPi) specimens. The results revealed that TCAB1 was overexpressed in approximate 80% NPC biopsies (40 out of 50 samples), but only in 15% of NPi biopsies (3 out of 20 samples) (Figure 1c, d). These data demonstrate that TCAB1 is overexpressed in human head and neck carcinomas, which suggests that TCAB1 may play an important role in head and neck cells tumorigenesis.

The previous studies revealed that TCAB1 overexpression might correlated with poor prognosis in head and neck cancers [16]. Although the samples of NPC patients were collected from 2011 to 2012, and most of them are less than 3 years, we still would like to investigate the correlation between TCAB1 overexpression and patient survival. Because of the time, we can’t do the 3 years survival curve, but we calculated the Median Survival Time based on our clinical follow-up survey. In all of the 50 NPC patients, 5 of them were dead up to now. The follow-up survey data showed that the median survival time is 27 months and the 95% confidence interval is 16.265-37.735.

### TCAB1 facilitates proliferation in head and neck carcinoma cell lines

**Figure 2:**
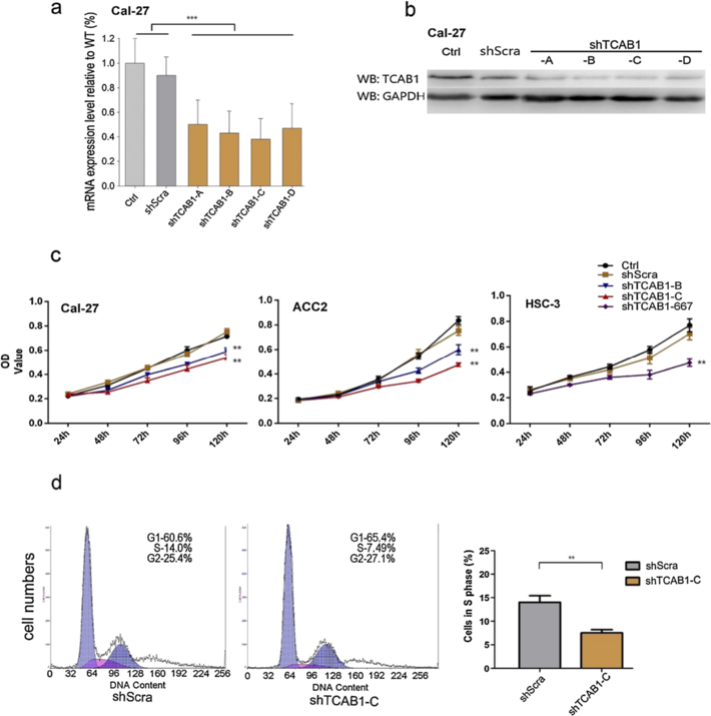
**Depletion of TCAB1 reduced the cell proliferation *in vitro*. a**. qPCR results. Using exogenous shTCAB1 lentivirus (4 different shRNA targets) depleted TCAB1 mRNA significantly. **b**. Protein level also significantly decreased after shTCAB1 lentivirus treatment. **c**. Depletion of TCAB1 by exogenous shRNA decreased cell proliferation in Cal-27, ACC2 and HSC-3 cells. **d**. FACS results. Depletion of TCAB1 might facilitate cancer cell arrest, and shTCAB1-treated cells are significantly less in S phase. Statistical analysis was determined by Student’s *t* test (**P < 0.01, ***P < 0.005).

Endogenous TCAB1 in OSCC cell lines Cal-27, HSC-3 and ACC cell line ACC2 was depleted using lentivirus carrying shTCAB1 plasmids. Quantitative PCR results show that TCAB1 decreased efficiently upon infection with lentivirus containing 5 different shTCAB1 sequences. It was also notable that the shRNA containing scramble sequence (shScra) had no effect as compared to the high knockdown efficiency of shTCAB1 (Figure 2a). Moreover, the western blot results indicated that TCAB1 drastically decreased in cells treated with shTCAB1 lentivirus (Figure 2b). The cells proliferation assay results demonstrated that cells treated with shTCAB1 lentivirus exhibited lower proliferation potential than the wildtype and shScra cells (Figure 2c), which is consistent with the previous study [16]. To avoid the experimental error, we repeated the CCK-8 assay at least twice independently and used different shRNAs and cell lines. Our studies display that TCAB1 is required for cells proliferation *in vitro*, and reduction of TCAB1 expression using exogenous shRNA leads to a corresponding decrease in their proliferation potential.

### Depletion of TCAB1 might induce cell cycle arrest

The data from CCK-8 assay showed that depletion of TCAB1 in Cal-27 reduced its proliferation potential. To verify this, we performed Fluorescence Activated Cell Sorter (FACS) experiment. The results also indicated that the cells treated with shTCAB1 lentivirus in S phase were sufficiently lesser than shScramble cells (Figure 2d). Although the underlying mechanism is still not understood, the study suggests that knockdown of TCAB1 *in vitro* influences the proliferation of OSCC cells.

### TCAB1 might influence invasion abilities of head and neck carcinoma cells *in vitro*

**Figure 3:**
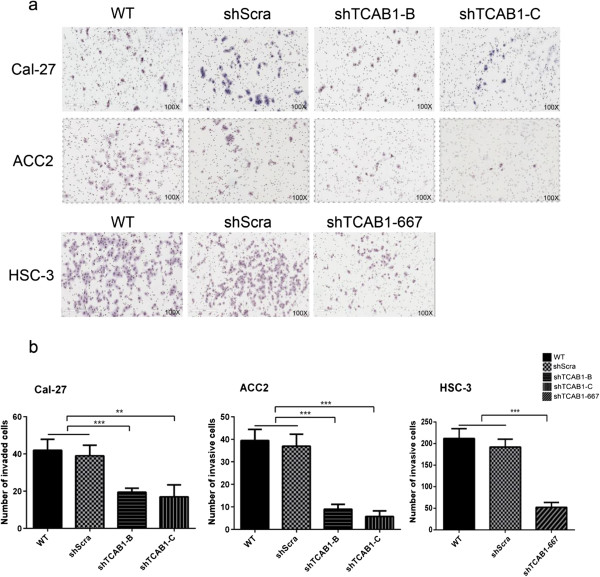
**Depletion of TCAB1 resulted in decreased cell invasion potential. a**. Depletion of TCAB1 in Cal-27, ACC2 and HSC-3 cells caused less cells invading through the micropores by trans-well test. **b**. Statistical analysis of the invaded cells, which was determined by Student’s *t* test (**P < 0.01, ***P < 0.005).

Invasion is one of important indicators of malignant tumors, and therefore, we explored whether TCAB1 is involves in cell migration process. Using the OSCC cell lines Cal-27, HSC-3, and ACC cell line ACC2 treated with shTCAB1 lentivirus, we tested the migration potential using matrigel-based trans-well experiments. The data indicated that knockdown of TCAB1 in Cal-27, ACC2 and HSC-3 significantly reduced the migration potential compared to negative control cells, the wildtype cells and shScra cells (Figure 3). Our studies suggest that TCAB1 indeed influence and facilitate head and neck carcinoma cells invasion *in vitro*.

### The oncogenic potential of TCAB1 may influence many pathways associated with cancer

To uncover which pathways were involved in TCAB1-mediated cancer regulation processes, we performed cDNA microarray analysis in an effort to identify some candidate pathways or factors. By comparing the expression level of genes between Cal-27 cells with and without shTCAB1 lentivirus infection, we selected thousands of differentially expressed genes (a 1.3-folds change was set as thresholds). Using KEGG pathway analysis tool online, we analyzed all the genes showing differences in expression, and the results showed that the TCAB1-regulated genes were involved in many pathways associated with cancers (Table 1). This included the p53 signaling pathway, the PI3K-AKT signaling pathway, the cell cycle and apoptosis pathway, Jak-STAT signaling pathway and so on. Here, we list the genes involved in Table 1. These studies reveal that TCAB1 may influence many cellular processes and pathways in cancer, but identification and confirmation of the exact pathway or factors is extremely difficult from the current microarray data.

### TCAB1 promotes tumor growth *in vivo*

**Figure 4:**
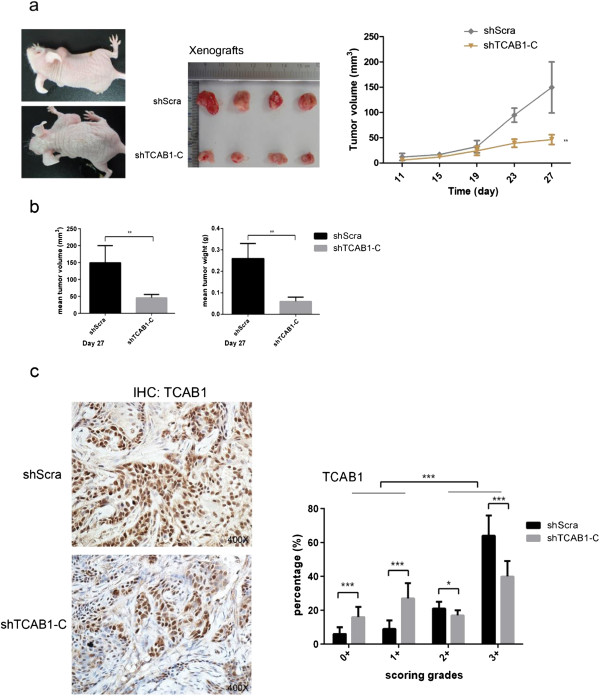
**TCAB1 knockdown inhibits tumor growth *in vivo*. a**. shTCAB1-C and shScra cells were injected subcutaneously into the left and right neck of the nude mice respectively. Measured the tumors size from the 11^th^ day using vernier caliper and calculated the volume of tumors by formula l*w*h/2 (mm^3^). Finally, got out the xenografts carefully after mercifully killed the mice. **b**. Measured the final volume and weighted the final weight of the removed tumors. **c**. Performed IHC against TCAB1 using mice xenografts sections. The smaller tumors expressed less TCAB1 compared to shScra cells. Statistical analysis of the IHC data used Aperio ImageScope software and all of the data were determined by Student’s *t* test (*P < 0.05, **P < 0.01, ***P < 0.005).

To further evaluate the oncogenic potential of TCAB1 *in vivo*, xenograft experiments were established in nude mice using OSCC Cal-27 cells with and without TCAB1 knockdown. The volume of tumors was tested every 4 days from the 11^th^ day when the tumors were tangible, and the data showed that the tumors generated from cells with TCAB1 knockdown grew slower compared to the scramble control (Figure 4a). Both the volume and the weight of the tumors were significantly less, which might result of the cells with TCAB1 knockdown had lower proliferation or growth potential compared to the shScra cells (Figure 4b). Fixed the xenografts tumor with formalin and embedded with paraffin, we performed IHC to check the TCAB1 expression of xenografts *in vivo*. The results showed the smaller generated tumors from shTCAB1-C cells expressed less TCAB1 relative to xenografts generated from shScra cells (Figure 4c).

### The decreased proliferation potential and increased apoptotic trend of shTCAB1 cells result in forming smaller xenografts tumor *in vivo*

**Figure 5:**
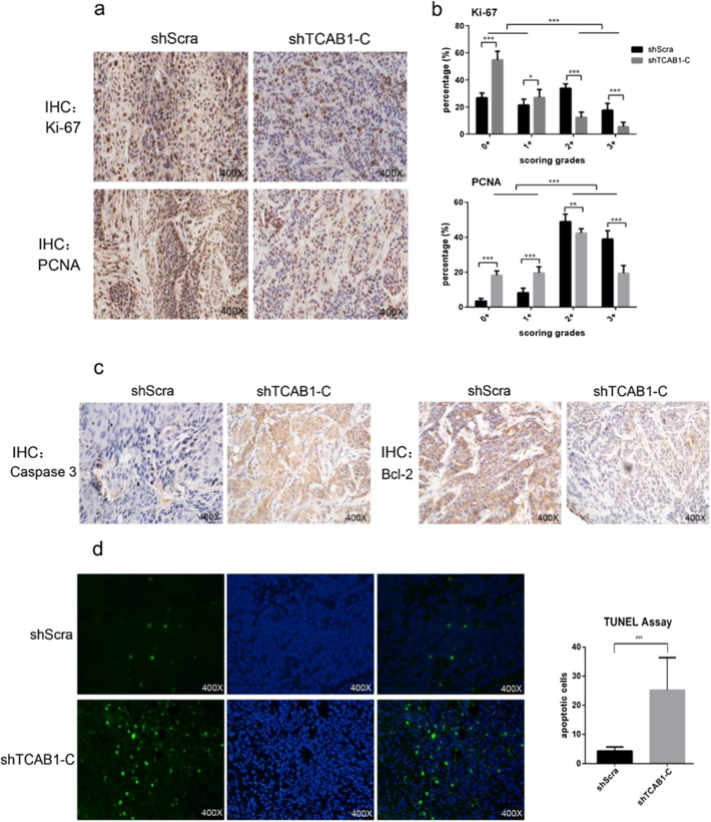
**Decreased proliferation potential and increased apoptotic trend due to depletion of TCAB1 resulted in smaller xenografts tumor *in vivo*. a**. Performed IHC against proliferation markers Ki-67 and PCNA using mice xenografts section, and the sections from shTCAB1 cells displayed lower expressed Ki-67 and PCNA. **b**. Statistical analysis of the Ki-67 and PCNA using Aperio ImageScope software. **c**. Apoptotic markers Caspase 3 and Bcl-2 were performed using IHC on mice xenografts sections. In xenografts derived from shTCAB1 cells, the results showed up-regulated Caspase 3 and down-regulated Bcl-2, the statistical analysis data were not shown here. **d**. TUNEL assay. Analyse the apoptosis of xenografts sections using TUNEL assay. Results indicated apoptosis was significantly increased in shTCAB1 cells. Statistical analysis of the data were determined by Student’s *t* test (*P < 0.05, **P < 0.01, ***P < 0.005).

**Figure 6:**
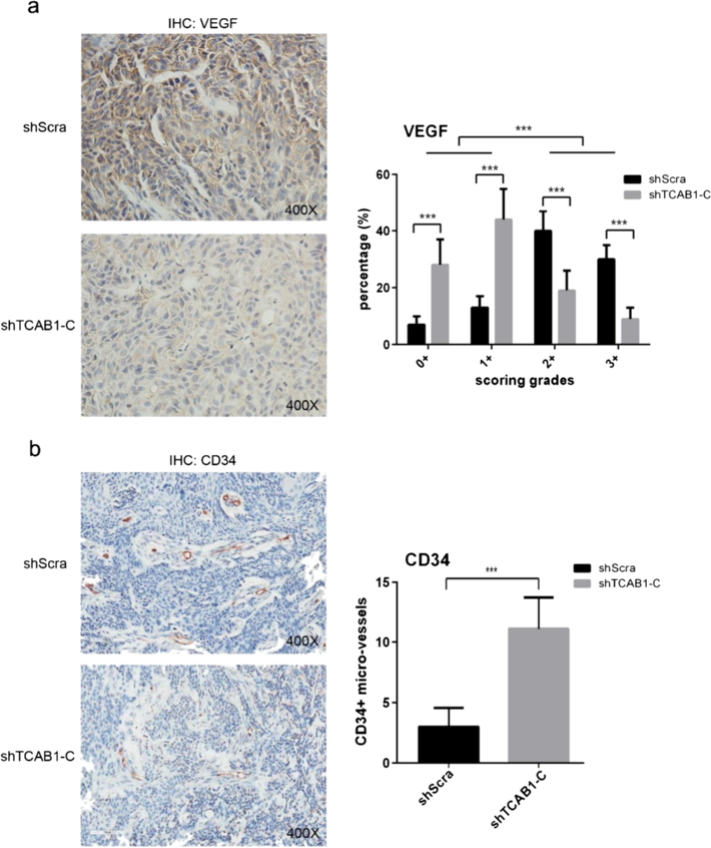
**The angiogenesis was inhibited in shTCAB1 xenografts. a**. IHC against VEGF was performed using mice xenografts sections. Statistical analysis of the VEGF expression used Aperio ImageScope software. Data showed the xenografts derived from shTCAB1 expressed less VEGF in cytoplasm. **b**. Performed IHC against CD34 using mice xenografts section. The results indicated xenografts from shTCAB1 displayed significantly less micro-vessels. Statistical analysis of the data were determined by Student’s *t* test (***P < 0.005).

In addition, we utilized the FFPE xenografts sections to investigate the reason of smaller tumors generated by shTCAB1-C cells *in vivo*. Firstly, we detected the expression of Ki-67 and PCNA, and the results indicated that the xenografts sections of shTCAB1-C cells expressed less than the other (Figure 5a, b), which suggested that the shTCAB1-C cells proliferation potential was also decreased *in vivo*. Meanwhile, we tested the apoptotic trend of cells with or without depletion of TCAB1 *in vivo* by some protein markers, like Caspase 3, Bcl-2, and TUNEL assay. The results indicated that in shTCAB1-C cells, the xenografts sections showed lower expressed Bcl-2 and upper expressed Caspase 3 (Figure 5c), which was consistent with the previous studies [16] and implied that the cells with TCAB1 depletion possessed higher apoptotic trend, resulting in more apoptosis cells. Otherwise, the TUNEL assay results also verified that sections from cells with TCAB1 depletion had more DNA fragmentations resulting of apoptotic signaling cascades (Figure 5d). Therefore, our data suggested that the decreased proliferation potential and the increased apoptotic trend might induce the cells growth much slower, resulting in generating smaller xenografts tumor *in vivo* finally.Besides, we wanted to know if the angiogenesis was influenced or influenced the tumor formation in the process. So we still performed IHC of VEGF and CD34 to check the micro-vessels generation in the xenografts. The results indicated that the sections from shTCAB1-C cells exhibited less VEGF expression in cytoplasm (Figure 6a). Otherwise, the micro-vessels displayed by CD34 signaling indicated that the more micro-vessels was necessary for supporting and supplying the bigger xenografts tumor formation (Figure 6b).

## Discussion

TCAB1, also known as WRAP53 isoforms β and γ, was involved in telomerase holoenzyme assembly and trafficking while the isoform WRAP53α was known as a natural antisense transcript that regulated p53 mRNA levels [10,11,15,23]. p53 is a very important suppressor gene in tumorigenesis as we all know, and a mutation in p53 may result in tumorigenesis. Furthermore, telomerase extends telomeres in most tumors, which assisted the prevention of senescence or apoptosis. Interestingly, WRAP53 plays important roles not only in stability of p53 mRNA, but also in telomerase holoenzyme assembly. These suggested that WRAP53 may have an effective role in two essential human tumorigenesis processes, namely the p53 pathway and the telomerase activity. All previously reported studies demonstrated that WRAP53 might be a major target molecule in cancer research, with possible functional relevance in head and neck carcinomas. In fact, WRAP53 itself has been the focus of investigation for many scientists. The following studies indicated that WRAP53 promoted cancer cell survival and depletion of WRAP53 using exogenous shRNA induces apoptosis *in vitro*[16], and also WRAP53 is necessary to form Cajal bodies and can direct the survival of motor neuron complex to Cajal bodies [24]. Cajal bodies were identified more than a century ago, and were seen as the site of initial modification and assembly of several U snRNPs [25,26]. While the function of WRAP53 based on Cajal body has been studied extensively, the mechanism of WRAP53 in tumorigenesis has also attracted much attention. Recently, Rao et al. demonstrated that WRAP53 was overexpressed in Esophageal Squamous Cell Carcinoma (ESCC) tissues compared to the adjacent non-neoplastic tissues [27]. However, there are so less and unclear studies on the functional significance of WRAP53 in head and neck cancers research thus far, and it would be interesting and beneficial to combine the knowledge on WRAP53 from previous studies and explore the possibility of its clinical application in head and neck cancers.

In our study here, we focused on the function of TCAB1, the WRAP53 isoforms except α, in tumorigenesis and development of head and neck carcinomas, so we used the symbol TCAB1 here but WRAP53.

Our studies showed that TCAB1 was overexpressed in three different head and neck cancer cell lines, including NPC, OSCC and ACC, as well as the clinical patient specimens sections detected by IHC. Depletion of TCAB1 using shRNA lentivirus can reduce the growth rate not only *in vitro*, which is consistent with previous study [16], but also *in vivo*. The results of CCK-8 assay exhibited the proliferation potential was significantly decreased in TCAB1 knockdown cells. Besides, the FACS data showed cells treated with shTCAB1 lentivirus were arrested in cell cycle, while the cells in S phase were significantly less than the shScramble cells, which meant that DNA replication in shTCAB1 cells was slower than in the control cells. All of these indicated that TCAB1 promotes cancer cell proliferation and a depletion of endogenous TCAB1 by exogenous shRNA reduces tumor growth rate *in vitro*. Furthermore, invasion is a very important parameter for malignant tumors. Hence, we performed an invasion test *in vitro* using trans-well experiments, and our results showed that depletion of TCAB1 reduced the invasion potential of head and neck cancer cells. It would be very interesting to study how TCAB1 facilitates cancer cells invasion in future.

Based on the proliferation and invasion test *in vitro*, we attempt to investigate the mechanism implicated in the process using cDNA microarray, which was performed on the platform of Affymetrix GeneChip Human Gene 1.0 ST Array. Almost thirty thousand genes’ transcription were tested in this assay. By comparing the expression level of every gene from two groups respectively, and setting 1.3-fold change as threshold, we indentified approximately 3.3 thousand differentially expressed genes. All of the differently expressed genes were analyzed online with Kyoto Encyclopedia of Genes and Genomes (KEGG) database to obtain the pathway maps [28]. The pathway distribution showed that several pathways associated with cancers were implicated in this process, just like the p53 pathway, cell cycle, apoptosis pathway and so on (detail in Table 1). Our data displayed that depletion of TCAB1 would affect the survival status of cancer cells, influence the proliferation and apoptosis. Therefore, the involvement of these pathways was not surprising at all. However, the cDNA microarray was performed to explore some candidate pathways or proteins which were regulated or mediated by TCAB1 or WRAP53. For instance, given p53 is an important cancer suppressor, the p53 pathway might by triggered by knockdown of TCAB1. The microarray data indicated many factors were up-regulated, including ATM, ATR, CDK1, CDK6, CCNB1, CCNB2, MDM4, SESN1, SESN3, ZMAT3, and FAS, while some genes were down-regulated, including GADD45A, GADD45B, CCND2, BBC3, IGFBP3, SERPINE1, and TP53AIP1. These data might give a direction for the further research. However, the current data are not sufficient to support an in-depth hypothesis. Depletion of TCAB1 might trigger many pathways directly or the changed cellular status might trigger these pathways indirectly. This needs further researches.

Xenografts assay in nude mice is very important for verifying the function of a protein in tumorigenesis or development. Our studies of the xenografts indicated that the shTCAB1 cells would form much smaller tumors *in vivo.* And through detecting many proliferation and apoptosis markers by IHC using the xenografts sections, we could find that the smaller xenografts tumor derived from shTCAB1 cells resulted of decreased proliferation potential and increased apoptotic trend. The data *in vivo* is consistent with the studies *in vitro*, which is very important to the further investigation for the diagnosis or therapy.

Angiogenesis is a critical event in tumor growth *in vivo*. Vascular endothelial growth factor (VEGF) plays a core role in angiogenesis, and its function has been verified in head and neck cancers [29,30]. Our data from the IHC of xenografts exhibited that VEGF was lower expressed in sections derived from shTCAB1cells. However, only the down-regulated VEGF is not sufficient. CD34 is a marker for micro-vessels, and the abundant vessels are necessary for supplying the tumor growth. In our study, the smaller tumors sections derived from shTCAB1 cells displayed much less micro-vessels, and which may limit the growth of xenografts tumor *in vivo*.

In the future, we will focus on the detail mechanism of the TCAB1/WRAP53-mediated function in head and neck cancers. The data generated from *in vitro* and *in vivo* here give us a future direction. Moreover, our studies and others previous studies encourage us for the future investigation.

## Conclusions

In this study, the data showed that TCAB1 was overexpressed in clinical specimens as well as in carcinoma cell lines. Depletion of TCAB1 using shRNA lentivirus inhibits cell proliferation and invasion *in vitro*. More importantly, TCAB1 knockdown reduces the growth of tumors *in vivo*. Given the oncogenic feature of TCAB1 genes, it should be an interesting potential target as a clinical diagnostic biomarker and might be beneficial as a future therapeutic target for head and neck carcinomas.

## Competing interests

The authors declare that they have no competing interests.

## Authors’ contributions

In this study, YL, LYX, JL and QMC participated the study design, and CKS, XBL, LH, YPG, KW, ZTX, PZ, CLZ and XLK mainly performed the experiments and generated data. CL supplied the NPC specimens and collected part of the follow-up information of the NPC patients. CKS, YL analyzed the data, wrote the manuscript, and QY helped to edited it. All authors read and approved the final manuscript.
